# Prognostic Factors for Recovery of Vision in Canine Optic Neuritis of Unknown Etiology: 26 Dogs (2003–2018)

**DOI:** 10.3389/fvets.2019.00415

**Published:** 2019-11-22

**Authors:** Christoforos Posporis, Elsa Beltran, Mark Dunning, Irene Espadas, Sabrina Gillespie, Amy Teresa Barry, Annette Wessmann

**Affiliations:** ^1^Neurology/Neurosurgery Service, Pride Veterinary Centre, Derby, United Kingdom; ^2^Queen Mother Hospital for Animals, Royal Veterinary College, Hatfield, United Kingdom; ^3^School of Veterinary Medicine and Science, University of Nottingham, Loughborough, United Kingdom; ^4^Willows Veterinary Centre and Referral Service, Shirley, United Kingdom; ^5^Small Animal Teaching Hospital, School of Veterinary Sciences, University of Liverpool, Neston, United Kingdom

**Keywords:** meningoencephalitis, immune-mediated, blindness, MUE, MUO, neuro-ophthalmology, optic nerve

## Abstract

Optic neuritis (ON) is a recognized condition, yet factors influencing recovery of vision are currently unknown. The purpose of this study was to identify prognostic factors for recovery of vision in canine ON of unknown etiology. Clinical databases of three referral hospitals were searched for dogs with presumptive ON based on clinicopathologic, MRI/CT, and fundoscopic findings. Twenty-six dogs diagnosed with presumptive ON of unknown etiology, isolated (I-ON) and MUE-associated (MUE-ON), were included in the study. Their medical records were reviewed retrospectively, and the association of complete recovery of vision with signalment, clinicopathologic findings, and treatment was investigated. Datasets were tested for normality using the D'Agostino and Shapiro-Wilk tests. Individual datasets were compared using the Chi-squared test, Fisher's exact test, and the Mann-Whitney U-test. For multiple comparisons with parametric datasets, the one-way analysis of variance (ANOVA) was performed, and for non-parametric datasets, the Kruskal-Wallis test was performed to test for independence. For all data, averages are expressed as median with interquartile range and significance set at *p* < 0.05. Twenty-six dogs met the inclusion criteria. Median follow-up was 230 days (range 21–1901 days, mean 496 days). Six dogs (23%) achieved complete recovery and 20 dogs (77%) incomplete or no recovery of vision. The presence of a reactive pupillary light reflex (*p* = 0.013), the absence of fundoscopic lesions (*p* = 0.0006), a younger age (*p* = 0.038), and a lower cerebrospinal fluid (CSF) total nucleated cell count (TNCC) (*p* = 0.022) were statistically associated with complete recovery of vision. Dogs with I-ON were significantly younger (*p* = 0.046) and had lower CSF TNCC (*p* = 0.030) compared to the MUE-ON group. This study identified prognostic factors that may influence complete recovery of vision in dogs with ON. A larger cohort of dogs is required to determine whether these findings are robust and whether additional parameters aid accurate prognosis for recovery of vision in canine ON.

## Introduction

Optic neuritis (ON) is a general term describing the pathological process of optic nerve inflammation and represents a clinical syndrome more than a specific disease (1, 2). Clinical signs are typically characterized by acute, bilateral or unilateral, partial or complete loss of vision (1, 3). Papillitis and neuroretinitis can be detected on fundoscopic examination (3–5) while retrobulbar ON generally lacks ophthalmoscopic abnormalities (1, 6, 7). Classification focuses on the underlying etiology in veterinary medicine. Presumed immune-mediated (5–13), neoplastic (1, 5, 14–19) and infectious diseases (5, 20–34) have been reported in association with ON. Other causes include extension of inflammation/infection and neoplasia from neighboring anatomical structures (1, 8, 14, 35), trauma (36), and toxins (5, 37, 38).

Canine ON of unknown etiology is believed to be immune-mediated. It is traditionally attributed to a focal form of granulomatous meningoencephalomyelitis (GME) or is considered part of a multifocal central nervous system (CNS) lesion-distribution of the same disease (6, 9, 10, 14, 39–42). Optic neuritis has also been reported in a dog with necrotizing leukoencephalitis (NLE) (1, 43). Both GME and NLE fall under the same umbrella term “meningoencephalomyelitis of unknown etiology (MUE)” but comprise disorders with clinicopathologic differences (11, 44–50). Definitive diagnosis requires histopathological examination (11, 44, 47, 51–53). In people, several pathogenetic theories have been proposed, and ON has been subdivided in typical and atypical forms (2). Optic neuritis is usually ascribed to multiple sclerosis (MS), and isolated forms are considered potential precursors of this disease (54). Other atypical causes of immune-mediated ON include neuromyelitis optica (NMO), neuromyelitis optica spectrum disorders (NMOSD), acute disseminated encephalomyelitis (ADEM), chronic recurrent immune optic neuropathy and autoimmune diseases with secondary optic nerve involvement (2, 55). These subtypes are characterized by distinct clinical courses and therapeutic requirements (2). Similarly, variations in the clinicopathologic presentation and outcome have been reported in dogs with idiopathic ON (5). There is currently no information available to classify canine ON cases as different phenotypic variants/stages of the same etiopathogenic entity or separate diseases.

This study focuses on canine ON of presumed immune-mediated etiology including isolated and MUE-associated forms. The clinicopathologic presentation, advanced imaging findings, treatment, and outcome in dogs with ON have been previously described (1, 3–12, 16, 39, 40, 56). While prognostic factors have been reported in dogs with MUE, such factors have not been specifically investigated with a focus on ON (10, 57–60). The purpose of this study is to identify prognostic factors for recovery of vision in canine ON. The authors hypothesized that a shorter duration of blindness prior to immunosuppressive therapy and a lower cerebrospinal fluid (CSF) total nucleated cell count (TNCC) are associated with complete recovery of vision.

## Materials and Methods

This study followed a retrospective multi-center design. The clinical records of dogs diagnosed with ON were reviewed. The database of three referral veterinary hospitals was accessed from 2003 to 2018: Pride Veterinary Centre; Queen Mother Hospital for Animals, Royal Veterinary College; Small Animal Teaching Hospital, University of Liverpool. Ethical approval was granted at all institutions involved.

Dogs of any age, sex and breed were included if all of the following criteria were met: (a) physical and neurological assessment at presentation and follow-up examinations at least 21 days apart; (b) presence of visual disturbance assessed by a neurology and/or ophthalmology clinician (resident, residency-trained or board-certified) based on the ability to navigate successfully obstacles; (c) advanced imaging of the head (CT or MRI) classified as adequate in terms of sequence/plane selection and diagnostic quality and interpreted by a board-certified neurologist and radiologist; (d) fundoscopic and/or advanced diagnostic imaging findings consistent with ON; (e) a maximum of 3-week history of visual disturbance; (f) absent/incomplete menace response and/or absent/incomplete pupillary light reflex (PLR) in the affected eye/s that could not be explained by other clinicopathologic or imaging findings. Cases were excluded if one or more of the following criteria were met: (a) intracranial or extracranial neoplasia (suspected or confirmed); (b) ocular inflammation or infection; (c) presumed toxin exposure or adverse reactions to medications; (d) systemic or CNS infection suspected based on positive serological and/or CSF screening tests; (e) suspected or witnessed ocular and/or orbital trauma; (f) incomplete clinical records at presentation or follow-up examinations; (g) follow-up period <21 days.

Data retrieved from medical records included patient signalment (age, sex, breed, neuter status), clinical history, presenting complaint, clinical, neurological and ophthalmologic examination, therapeutic protocol, and outcome. Further diagnostic tests included CBC, serum biochemistry profile, urinalysis, electroretinography (ERG), CSF analysis, and screening tests for infectious diseases. Protein concentration in CSF >27 mg/dl for cerebellomedullary and TNCC >5 cells/μl were classified as increased (61).

Fundoscopic findings were classified as consistent with ON when signs of papillitis and/or neuroretinitis were detected. Papillitis was characterized by a swollen optic disc with blurred margins, loss of the physiologic cup, raised blood vessels on the disc surface, hemorrhages and/or exudates involving the optic disc. Neuroretinitis was diagnosed when the extension of these pathological changes to the retina occurred (1, 3, 4, 7, 12).

Magnetic resonance imaging of the head was performed using four 1.5 Tesla Magnets (Intera and Ingenia CX, Philips Medical Systems, Eindhoven, the Netherlands; Siemens Magnetom Essenza, Frimley, United Kingdom; HDe, GE Healthcare, United Kingdom). Selected sequences included T2-weighted, Fluid-Attenuated Inversion Recovery (FLAIR), Short Tau Inversion Recovery (STIR), Gradient Echo, pre- and post-contrast T1-weighted, and post-contrast T1-weighted fat suppression images. Sagittal, dorsal, transverse and oblique dorsal and sagittal planes were evaluated. CT of the head was performed using a 16-slice CT scanner (Mx8000 IDT, Philips, Best, the Netherlands). Post-contrast images were acquired using Gadoterate Meglumine (Dotarem®, Guerbet, UK) for MRI and Iohexol (Omnipaque® 300, GE Healthcare, UK) for CT. Magnetic resonance imaging findings were assessed for optic nerve lesions consistent with ON as follows: presence of hyperintense signal on T2-weighted, FLAIR and STIR images; enhancement on T1-weighted post-contrast images and/or T1-weighted fat suppression post-contrast images; enlargement of the optic nerve or optic chiasm with no significant mass lesion; loss of the subarachnoid CSF signal of the intra-orbital optic nerve segment secondary to thickening of the optic nerve; optic nerve head swelling (7, 12, 13, 62, 63). Computed tomography findings of suspected ON consisted of the following abnormalities involving the optic nerve: contrast uptake; optic nerve head swelling; enlargement of the optic nerve or optic chiasm with no significant mass lesion (64–67).

Dogs were divided into two groups: presumptive MUE-associated ON with multifocal CNS disease (MUE-ON) and presumptive isolated ON (I-ON). Dogs with I-ON were characterized by the absence of clinical or advanced imaging findings involving the CNS outside the optic nerve. Dogs with MUE-ON had additional neurological deficits and/or advanced imaging findings involving the CNS outside the optic nerve. Group allocation did not depend on the results of CSF analysis.

Dogs receiving only corticosteroids were assigned in the monotherapy group and those treated with any additional immunosuppressive medication in the polytherapy group. The outcome was based on comparing the following parameters before and after treatment: the ability to navigate obstacles successfully, menace response, and PLR. The menace response was subjectively assessed as complete, incomplete, or absent. The PLR was classified as reactive (PLR present) referring to a complete or an incomplete reflex and non-reactive (PLR absent) referring to an absent reflex. Dogs were divided into two groups based on their visual outcome: (a) complete recovery of vision; (b) no or incomplete recovery of vision. Complete recovery of vision was characterized by the following: successful navigation of obstacles and normal vision reported by the owner; complete menace response in both eyes; complete PLR in both eyes. Incomplete recovery of vision was characterized by at least one of the following: partial improvement in navigating obstacles and partial improvement of vision reported by the owner; improved but incomplete menace response in at least one eye; improved but incomplete PLR in at least one eye. No recovery of vision was characterized by all of the following: no improvement of the ability to navigate obstacles and no improvement of vision reported by the owner; no improvement of the menace response with or without deterioration in either eye; no improvement of the PLR with or without deterioration in either eye. Dogs with no recovery of vision that further deteriorated as well as the prevalence of relapses in all dogs were recorded. Follow-up period was defined as the number of days from the first presentation until the final re-examination and communication with the owner and/or the referring veterinary surgeon. Follow-up data included clinical and neurological findings as well as telephone communications with owners and referring veterinary surgeons.

Statistical analysis was carried out using Graph Pad Prism 7.00 (GraphPad Software, La Jolla, California USA). Tests for normality were performed on each group (D'Agostino and Shapiro-Wilk Tests). Individual datasets were compared using the Chi-squared test, Fisher's exact test and the Mann- Whitney U-test. For multiple comparisons with parametric datasets, the one-way analysis of variance (ANOVA) was performed, and for non-parametric datasets, the Kruskal-Wallis test was performed to test for independence. Dunn's multiple comparisons test was used to analyze differences between specific groups. For all data, averages are expressed as median with interquartile range with significance set at *p* < 0.05.

## Results

### Case Selection

A total of 46 dogs with ON were identified. Nine cases were excluded due to insufficient follow-up (two dogs), inadequate clinical information (one dog), suspected infectious diseases (two dogs), suspected intracranial (three dogs), and extracranial (one dog) neoplasia. Eleven dogs did not meet the inclusion criteria due to the lack of advanced diagnostic imaging or negative MRI/CT and fundoscopy results. The remaining 26 dogs with ON fulfilled the inclusion criteria and were included in the study.

### Signalment

The median age at presentation was 47.5 months (range 7–132 months, mean 49.2 months). Males represented 58% of the population (15 dogs) and females 42% (11 dogs), with eight spayed female and 10 neutered male dogs. The most prevalent dog breed was French Bulldog with 11 cases (42%), followed by Jack Russell Terrier with three cases, Shih Tzu and Lhasa Apso with two cases each and one dog from the following breeds: Patterdale Terrier, Bedlington Terrier, Finnish Lapphund, English Springer Spaniel, Border Collie, Boston Terrier, West Highland White Terrier, and Cairn Terrier. The prevalence of French Bulldogs in the general hospital canine population of two participating referral centers providing 22 out of 26 cases was 1.7%. This information was not available from the third referral hospital. Median body weight at presentation was 10.8 kg (range 6–20.7 kg, mean 11.6 kg). Age at presentation had a significant effect on outcome (*p* = 0.038) (Table 1). The younger the dog was at presentation, the more likely a complete recovery of vision. No statistically significant association was identified between complete recovery of vision and breed, gender, neuter status, or weight.

**Table 1:** Clinicopathologic findings with prognostic value in 26 dogs with ON.

**Recovery of vision**	**Statistically significant prognostic factors**							
	**Age (months)**			**Dogs with reactive PLR (%)**	Dogs with fundoscopic lesions (%)^*^	**CSF TNCC (cells/μl)**		
	**Median**	**Mean**	**Range**			**Median**	**Mean**	**Range**
Complete recovery	21.5	26	7–62	4/6 (67%)	1/5 (20%)	1.5	4.5	0–20
Incomplete/no recovery	58.5	56.2	11–132	2/20 (10%)	18/18 (100%)	20	150	0–1,065
*p*-value		0.038^a^		0.013^b^	0.0006^b^		0.022^a^	

*PLR, pupillary light reflex; CSF, cerebrospinal fluid; TNCC, total nucleated cell count; μl, microliter*.

* *Fundoscopic examination was performed in 23 out of 26 dogs*.

a *Mann-Whitney test*.

b *Fisher's exact test*.

### Clinical Signs

Ten dogs (38%) had partial loss of vision while 16 (62%) were completely blind at presentation. One dog had enucleation of one eye before ON; thus, a total of 51 eyes were assessed out of which 48 were classified as affected. A reactive PLR was observed in 19 out of 48 affected eyes (40%; nine eyes with complete PLR, 10 eyes with incomplete PLR) while 29 eyes (60%) were characterized by a non-reactive PLR. Menace response was abnormal in 45 out of 48 affected eyes (94%) with an absent response in 43 and an incomplete response in two eyes. Except for two affected eyes in two different dogs, the menace response was always characterized by either the same or a worse degree of deficit compared to the PLR of the same eye at presentation. Visual deficits were predominantly bilateral (22 dogs; 85%). The median duration of blindness before immunosuppressive treatment was 6 days (range 1–21 days, mean 7.3 days) and the median delay of treatment (time from presentation to immunosuppressive treatment) was 1 day (range 0–15 days, mean 1.8 days). The presence of a reactive PLR (*p* = 0.013) at presentation was statistically associated with complete recovery of vision (Table 1). There was no statistically significant influence identified of the severity of menace response deficit, laterality of ON, severity of visual disturbance and duration of blindness before immunosuppressive treatment.

### Investigations

Infectious disease screening included CSF PCR for *Toxoplasma gondii, Neospora caninum, Borrelia burgdorferi, Ehrlichia canis, Ehrlichia ewingii, Anaplasma phagocytophilum, Anaplasma platys*, and *Canine Distemper Virus*; serum antibody titers for *Toxoplasma gondii, Neospora caninum, Leptospira spp*.; latex cryptococcal antigen test (LCAT) and in-clinic Idexx SNAP 4Dx Plus test (SNAP 4Dx Plus, IDEXX Laboratories, UK) for *Ehrlichia* canis, *Ehrlichia* ewingii, Borrelia burgdorferi, Anaplasma phagocytophilum and Anaplasma platys. All but five dogs were tested for infectious diseases and were negative.

Fundoscopic examination was performed in 23 cases (88%), and lesions consistent with ON were found in 19 out of 23 (83%). No abnormality was identified on fundoscopy in the remaining four dogs (17%). Electroretinography was performed in nine cases, including the four dogs with normal fundoscopy and was within normal limits. The absence of fundoscopic lesions was significantly associated with complete recovery of vision (*p* = 0.0006) (Table 1).

Advanced imaging of the head was performed in all cases (MRI in 25 dogs, CT scan in one dog). Lesions identified on advanced imaging were positive for ON in 21 dogs (81%). All five dogs with no optic nerve lesions on MRI/CT were diagnosed with MUE, but concurrent ON was suspected clinically and was confirmed on fundoscopy. Thirteen out of 19 dogs (68%) with MUE-ON showed no clinical signs referring to CNS disease outside the optic nerve on history or neurological examination. The absence of optic nerve lesions on MRI/CT was not associated with complete recovery of vision.

All dogs had cerebellomedullary CSF analysis performed. One case was excluded from further analysis of TNCC and protein concentration due to severe blood contamination. This case was classified as having normal CSF despite the iatrogenic blood contamination. For the remaining 25 cases, 17 (68%) showed abnormalities. Fourteen dogs (56%) had an increased protein concentration (median 30 mg/dl, mean 56 mg/dl, range 28–490) and 13 (52%) an elevated TNCC (median 20 cells/μl, mean 137 cells/μl, range 7–1,065). No statistically significant association was identified between CSF protein concentration and complete recovery. The CSF TNCC was significantly associated with complete recovery of vision (*p* = 0.022). The lower the TNCC the higher were the chances for complete recovery of vision (Table 1).

### Diagnosis

Diagnosis of presumptive ON was reached based on the identification of both fundoscopic and advanced imaging findings consistent with ON in 14 dogs (54%), advanced imaging findings alone in seven dogs (27%) and fundoscopic findings alone in five dogs (19%). The diagnosis was supported by the clinical/neurological examination findings, CSF analysis, ERG, and negative infectious diseases tests. Nineteen cases (73%) were diagnosed with MUE-ON and seven (27%) with I-ON. No association was found between the diagnosis of I-ON or MUE-ON and complete recovery of vision. Dogs with I-ON were significantly younger (*p* = 0.046) and had lower CSF TNCC (*p* = 0.030) compared to the MUE-ON group, which appeared to have significantly more neutered dogs (*p* = 0.014) (Table 2). Dogs with fundoscopic lesions consistent with papillitis and/or neuroretinitis were statistically more likely to have a non-reactive PLR (*p* = 0.040), complete loss of vision (*p* = 0.024), higher CSF protein concentration (*p* = 0.029) and TNCC (*p* = 0.033) compared to dogs with retrobulbar ON.

**Table 2:** Comparison of statistically significant clinicopathologic differences between dogs with MUE-ON and I-ON.

**Type of ON**	**Statistically significant clinicopathologic differences between groups**				
	**Median age (months)**	**Number of neutered dogs (%)**	**CSF TNCC (cells/μl)**		
			**Median**	**Mean**	**Range**
MUE-ON	59	16/19 (84%)	20	128	0–1065
I-ON	19	2/7 (29%)	2	82	0–560
*p*-value	0.046^a^	0.014^b^		0.030

*MUE-ON, meningoencephalitis of unknown etiology-associated optic neuritis; I-ON, isolated optic neuritis; CSF, cerebrospinal fluid; TNCC, total nucleated cell count; μl, microliter*.

a *Mann-Whitney test*.

b *Fisher's exact test*.

### Treatment

Twelve dogs had received treatment before referral, but none was immunosuppressed. No history of any medications given prior to referral was available in two cases. Eyedrops (Maxitrol, Pred Forte, Azopt) were used in five dogs, non-steroidal anti-inflammatories in six (meloxicam, carprofen, robenacoxib), a single anti-inflammatory dose of dexamethasone in one dog, antibiotics in three (amoxicillin-clavulanic acid, trimethoprim sulfadiazine), and chlorphenamine, paracetamol, tramadol, and imepitoin in one case each. The median duration of anti-inflammatory therapy before presentation in these seven cases was 3 days (range 1–14 days, mean = 5.4 days). Their CSF TNCC at presentation was normal in two (<5 cells/μl) and increased in five dogs (median = 41 cells/μl, mean = 116 cells/μl, range seven to 404 cells/μl). After diagnosis, all dogs received immunosuppressive therapy on a tapering regimen consisting of at least corticosteroids (prednisolone or dexamethasone followed by prednisolone). One or more adjunctive immunosuppressive medications were used in 22 dogs (85%) for at least one month: cytosine arabinoside (18 dogs), cyclosporine (four dogs), leflunomide (one dog), and lomustine (one dog). Antibiotics (clindamycin and doxycycline) were used briefly in three dogs and were discontinued once negative results from infectious diseases were received. No difference in outcome was identified between dogs treated with monotherapy or polytherapy.

### Follow-up, Outcome, and Relapses

Follow-up ranged from 21 to 1,901 days (median 230; mean 496). Complete recovery of vision was achieved in six out of 26 dogs (23%). Incomplete or no recovery of vision was recorded in 20 dogs (77%) with 11 (42%) showing signs of incomplete improvement and nine (35%) having no recovery. Only one out of seven dogs pre-treated with anti-inflammatory medications prior to referral achieved complete recovery of vision. No deterioration after immunosuppressive treatment was seen in any of the cases. Seven out of nine dogs with no recovery of vision were diagnosed with MUE-ON. While their vision remained unchanged, improvement of their MUE-associated clinical signs was reported. Nine dogs (35%) had at least one relapse from 102 to 827 days after the initial diagnosis of ON. Seven out of these nine dogs were diagnosed with MUE-ON and two with I-ON.

## Discussion

This study identified factors associated with visual recovery in 26 dogs with ON. A younger age, a reactive PLR, the absence of fundoscopic lesions, and a lower CSF TNCC at presentation were statistically associated with complete recovery of vision. To the best of our knowledge, no similar studies investigating prognostic factors in dogs with ON currently exist. In people with ON, prognosis mainly depends on the underlying subtype of ON, and diagnostic accuracy is highly relevant for approach to clinical management (68) (Table 3). Visual recovery is slightly worse in patients with MS-associated ON compared to those with isolated disease, but both have an overall positive long-term outcome (72). Atypical forms of ON such as NMO/NMOSD have a poorer prognosis and more recurrent attacks (68, 73–75). Severe loss of visual acuity at presentation (76, 77), specific ethnicity/race (78), late treatment (79), bilateral ON (80), involvement of the intra-canalicular part of the optic nerve on MRI, absence of retrobulbar pain (77), and older age at onset (76) were associated with poorer outcomes yet not all authors validated these results (78, 80).

**Table 3:** Severity and prognosis in human immune-mediated ON-associated diseases (68–71).

**ON-associated disease**	**Severity**	**Prognosis**
MS-associated ON Solitary (monophasic) isolated ON	Usually unilateral mild to moderate visual loss and mild periocular pain	Spontaneous improvement in >90% of patients
NMO-associated ON	Usually bilateral severe visual loss Transverse myelitis	Incomplete recovery Attacks of ON and/or transverse myelitis recur in >85% of patients with paralysis or blindness seen in 50% of cases within 5 years
Chronic relapsing inflammatory optic neuropathy	Usually bilateral severe visual loss with persistence of pain after onset of blindness	Steroid-responsive (long-term)Frequent relapses when treatment is reduced/interrupted
ADEM-associated ON	High incidence in pediatric population Encephalomyelitis with severe bilateral optic neuritis in up to 15% of patients.	Complete recovery in 56–94% Multiphasic ADEM in 0–23% Monophasic ADEM followed by recurrent ON in 0–9% Residual visual deficits are common

*ON, optic neuritis; MS, multiple sclerosis; NMO, neuromyelitis optica; ADEM, acute disseminated encephalomyelitis*.

The younger the dog was at presentation the higher were the chances for complete recovery of vision in this study population. Similarly, a recent publication investigating canine MUE showed that younger age at onset was associated with longer survival, but none of these dogs were diagnosed with concurrent ON (58). Still, other authors failed to repeat this finding (11, 57). In human medicine, pediatric ON is characterized by better recovery of visual acuity and lower annual relapse rates (76, 77, 81–83). In contrast, younger-onset patients with NMO from the United Kingdom have a higher risk for visual disability (73). Interestingly, dogs with I-ON were significantly younger compared to those with MUE-ON. Considering the correlation between human medicine data and our results, a specific pediatric ON phenotype could be considered in young dogs. The question also arises whether aging influences recovery and remyelination of the optic nerves or if benign phenotypes of ON are more common in younger dogs. Generally, remyelination decreases with age, as seen with any other regenerative process (84). Activation, recruitment, and differentiation of oligodendrocyte progenitor cells (OPC) decline with age (85–90). In addition, regulation of OPC behavior is influenced by signaling molecules secreted by macrophages or by phagocytized myelin debris containing inhibitors of OPC differentiation (91, 92). These processes are delayed with age (89, 93). Myelin debris clearance and remyelination are greater in younger animals due to more performant circulating monocytes (84). In clinical practice, the influence of aging could have an impact on demyelinating diseases such as ON.

A high number of French Bulldogs (42%) was recorded in our study. The popularity of the breed could have biased this finding but the prevalence of French Bulldogs in the general canine population of the referral hospitals providing 22 out of 26 cases was only 1.7%. While MUE/ON represents 25% of encephalopathies in French Bulldogs (94), no data on familial inheritance is currently available in this breed. In people, genetic factors play a role in the development of MS and NMO, both of which are associated with ON but have significantly different prognosis (95–97). Interestingly, the strongest genetic risk in MS is related to the human leukocyte antigen (HLA) class II—DRB1 region which reflects similarities with the canine NME model association to the dog leucocyte antigen class II (DLA II) (98–101). Another common functional genetic variant between dogs and people was found outside the HLA II region, within the IL7R gene (96, 101). Most of the French Bulldogs included in our study (73%) were diagnosed with presumptive MUE, but the lack of histopathological confirmation prevented further categorization into NME or other types of MUE. The identification of predisposing genetic factors could achieve a more specific classification of canine ON sub-types. However, it is currently unclear if this could also result in prognostic contributions. Comprehending the differences in outcome and clinical course between distinct human ON-associated disorders, one can also suspect the clinical relevance of a potential sub-categorization of canine ON in achieving accurate prognostication. The association between genetic predisposition and prognosis remains to be explored in future studies.

The impression that ON requires immediate immunosuppressive treatment predominates among practitioners. Surprisingly, the duration of blindness prior to immunosuppressive therapy was not statistically associated with the visual outcome in our study population; thus, one of our hypotheses was rejected. This finding can have clinical relevance in the decision-making process for the treatment of canine ON. One of the most common dilemmas clinicians face during this process is whether to start treatment immediately or to wait for results of infectious disease testing. Indeed, knowing if delaying treatment influences the prognosis or not can have an impact on clinical decision-making. In our study population, the six dogs with complete recovery of vision received treatment 2 days (1 case), 7 days (2 cases), 12 days (1 case), 13 days (1 case), and 21 days (1 dog) after onset of blindness but still achieved complete recovery of vision. The possibility that treatment prior to presentation could have influenced the outcome by improving the chances of recovery was considered. However, only one out of seven dogs that were pre-treated with anti-inflammatory medications before referral achieved complete recovery of vision. Moreover, our data of seven pre-treated dogs showed elevation of CSF TNCC in five dogs, and thus it could not be concluded that pre-treatment prior to presentation has a protective effect or influence on the final outcome. Immunosuppressive medications were not used in any of the included cases prior to diagnosis of ON. Although our results suggest that duration of blindness prior to treatment does not appear to influence outcome, these findings require validation by larger sample size studies and changes in current practice cannot be recommended at this stage. Furthermore, the maximum duration of blindness prior to immunosuppressive treatment was 3 weeks, and this limited our ability to investigate the prognosis in dogs with longer treatment delay. Contrariwise, considering that factors such as presence of PLR, normal fundoscopy, and lower CSF TNCC were associated with complete recovery of vision, it is possible that parameters related to the severity of the disease have more prognostic value than its actual duration. In people with ON, one study reported improved visual acuity in Thai patients with isolated ON treated within 8 days from onset (79). In another study, early treatment (within 7 days from onset) was associated with better short-term visual outcomes in people with severe acute ON, but long-term visual acuity was not influenced by the timing of treatment (102).

The presence of PLR was associated with complete recovery of vision in this study. The PLR can be a marker of optic nerve dysfunction (103). In people, quantitative PLR assessments show significantly decreased results in acute ON (104) and correlate with the severity of damage (105, 106). However, qualitative PLR examination is a subjective evaluation of the degree of pupillary constriction, which depends on the experience of the examiner and light intensity (107, 108). Our results showed that the presence of PLR is not uncommon among dogs with ON with 40% of affected eyes having a reactive PLR. Similarly, reactive PLR (incomplete or intact) was previously reported in 21% of eyes with immune-mediated ON (5). Hence, the presence of PLR should not drive clinicians away from the neuroanatomical diagnosis of optic neuropathy.

The absence of fundoscopic lesions was associated with complete recovery of vision. As long as other pathognomonic features of ON are present and primary retinal pathology is ruled out, the lack of fundoscopic abnormalities is indicative of retrobulbar ON (1, 7). Based on previous reports (5, 7) and in corroboration with our data, it appears that retrobulbar ON has a low prevalence in dogs. In people, retrobulbar ON occurs in two-thirds of patients with typical ON (109). In our study, dogs with papillitis/neuroretinitis were statistically more likely to have a non-reactive PLR, complete loss of vision, higher CSF protein concentration and TNCC. It is possible that the involvement of the optic disc/retina is associated with more severe or irreversible lesions compared to a retrobulbar localization. We could also speculate that retrobulbar ON represents a less aggressive ON phenotype characterized by better visual outcomes. However, the absence of fundoscopic lesions is not a specific feature of certain ON phenotypes in people (75, 110). In addition, optic disc swelling accompanied by mild initial visual acuity loss was associated with better short-term outcomes in people with bilateral acute ON, and this somewhat contradicts our findings (80).

A lower CSF TNCC was associated with complete recovery of vision in our study. Five out of six dogs with complete recovery of vision had a normal CSF TNCC, and these findings support one of our hypotheses. Smith and colleagues reported abnormal CSF analysis in 41 and 91% of dogs with MRI/CT-confirmed I-ON and MUE-ON, respectively. Similar to our findings (Table 2), this study reported that the CSF TNCC was significantly higher in dogs with MUE-ON (median = 146 cells/μl) than in those with I-ON (median = five cells/μl) (5). In canine MUE, a higher CSF TNCC was associated with shorter survival (58), whereas this finding was dismissed by an earlier study (11). In people, the presence of CSF pleocytosis is not a pathognomonic feature of specific ON subtypes, but a greater pleocytosis is expected with NMO than MS (111–113). The degree of pleocytosis could correspond to the severity of inflammation and subsequent damage which then could have an impact on visual outcome. However, the significance of pleocytosis in canine immune-mediated CNS diseases, including ON, is currently unknown. Interestingly, dogs with steroid-responsive meningitis arteritis can present with prominent pleocytosis but prognosis for recovery remains excellent with appropriate therapy (114–116), whereas up to 57% of dogs with MUE can have normal CSF cytology (48, 117) and this does not necessarily reflect a better prognosis (11, 57, 60).

In search of prognostic factors in human ON, the value of serum/CSF biomarkers in the classification of this syndrome in pathogenetically distinct phenotypes cannot be underestimated. Accurate diagnosis and differentiation between ON sub-types have been facilitated by the discovery of such markers (118–122) (Table 4). This is closely related to prognosis due to their well-known differences in outcome, clinical course, and response to various therapies (2, 68–71, 140, 141). Even within the same ON phenotype, specific biomarkers have been demonstrated to predict the clinical course of the disease. For example, oligoclonal immunoglobulin G and M bands were shown to predict the onset of conversion of isolated ON to MS (123). In veterinary medicine, disease-specific markers and their potential prognostic value have not yet been validated. Auto-antibodies against astrocytes such as anti-glial fibrillary acidic protein (GFAP) (142, 143) and anti-transglutaminase-2 (TG-2) antibodies (144) have been identified in dogs with MUE and particularly NME. Nonetheless, there is currently no evidence available linking the presence of these biomarkers to canine ON and its outcome.

**Table 4:** Biomarkers with prognostic value in people with ON (72, 81, 112, 118–139).

**Biomarker**	**Contribution in diagnosis**	**Prognostic value**
*AQP-4* Ab	Positive in NMO Negative in MS Distinction of NMO from MS with a sensitivity of 68–91% and specificity of 85–99%	More severe and relapsing ON Higher rate of conversion to NMO within 1 year Poorer visual outcomes compared to other phenotypes
*OCB*	Positive in 20–30% of patients with NMO, 6–13% in MOGAD and 80% in MS.	Spontaneous recovery in MS-ON Lipid-specific oligoclonal IgM bands could indicate a more severe clinical course and predict earlier onset of MS
*MOG Ab*	Diagnosis of MOGAD Rare in adults with MS Absent in AQP-4-seropositive NMO patients Seropositivity in children with AQP-4-seronegative NMOSD, recurrent ON, transverse myelitis and multiphasic or monophasic ADEM	Similar to NMO phenotype but better visual outcomes reported Negative and positive clinical courses reported Recurrent attacks of ON
*Gly-R Ab*	Currently unknown Positive in adults with ON	Relapsing isolated ON
*NMDA-R Ab*	NMDA-R encephalitis Positive in some adults with ON	Monophasic episodes of NMOSD, brainstem or multifocal demyelinating syndromes
*GFAP*	Astrocytic biomarker of highly active inflammation in NMO GFAP concentration is believed to be higher in NMO than in MS	Correlation with clinical severity was demonstrated between CSF GFAP and NMO relapses but also between serum GFAP and relapsing-remitting MS
*Interleukin-6 Interleykin-6-receptor*	Notable increases in CSF interleukin-6 and soluble interleukin-6 receptor concentration identified in NMO	Currently unknown
*MRZ reaction*	Positive in MS (78%) Rare in most patients with atypical types of ON such as NMO, ADEM and autoimmune encephalitis	Spontaneous recovery in MS-ON

*AQP-4, aquaporin 4; MOG, myelin oligodendrocyte glycoprotein; MOGAD, MOG-IgG-associated disorder; Ab, antibody; Gly-R, glycine receptor a1 subunit; NMDA-R, N-methyl-D-aspartate receptor; OCB, oligoclonal bands; NMOSD, neuromyelitis optica spectrum disorders; NMO, neuromyelitis optica; MS, multiple sclerosis; ADEM, acute disseminated encephalomyelitis; ON, optic neuritis; GFAP, glial fibrillary acidic protein; MRZ, measles, rubella and varicella zoster virus*.

The presence of optic nerve lesions on MRI/CT was not associated with the outcome in this study. While MRI appeared to have high sensitivity in identifying lesions suggestive of ON, 19% of included dogs had no MRI lesions of the optic nerve. This has been previously described in dogs with ON (5), and a normal MRI was also reported in 24% of dogs with inflammatory CSF (145). The overall agreement between neurological examination and MRI findings can be as low as 60% (48, 146). Likewise, subclinical brain lesions were identified in 68% of our patients with MUE-ON. This demonstrates the limited sensitivity of the clinical assessment in identifying mild encephalopathic signs in visually impaired dogs (48, 146). It further highlights the importance of MRI in the diagnosis of ON-associated diseases as the presence of parenchymal brain lesions may have an impact on the general prognosis and survival. Moreover, MRI is superior compared to CT in detecting such lesions. Thus it cannot be excluded that the one dog with CT-confirmed I-ON could have been misclassified as brain parenchymal lesions might have been missed.

Dogs with ON are traditionally treated with long-term immunosuppressive protocols. This is not a surprise considering the potential life-threatening implications of MUE and its frequent association with ON. In human patients, therapeutic requirements vary depending on the underlying etiology. In MS-associated ON, faster recovery can be seen with corticosteroids, but the long-term outcome is positive even without immunosuppressive therapy (76, 147). Chronic relapsing inflammatory optic neuropathy and NMO require high-dose and prolonged corticosteroid treatment (2). The investigation of canine ON phenotypes not requiring immunosuppression is highly problematic due to ethical considerations of not treating a condition that is frequently associated with MUE.

Visual outcome was generally poor in this study as only 23% of included dogs achieved significant visual recovery. Similar results were reported by Smith and colleagues, with only 17% of dogs with ON achieving complete recovery of vision (5). Recurrence was recorded in 35% of our cases and is a known phenomenon in canine MUE and ON (5, 9, 12, 39). In people, recurrence occurs in 28% of patients within 5 years and 35% within 10 years (148, 149). The concurrent diagnosis of underlying diseases such as NMO and MS, the presence of unilateral ON and the use of relatively low initial glucocorticoid dosage were associated with higher recurrence rates (150). In our study, the concurrent diagnosis of MUE and the laterality of ON did not have an impact on visual outcomes. The association of different parameters with relapsing disease was not statistically investigated.

Limitations of this study are its retrospective multicenter nature and small sample size, especially of the complete recovery group. Fundoscopic examination was not performed in all dogs and this inevitably decreased the total number of cases used for statistical evaluation of fundoscopy as a prognostic factor. All 4 cases with normal fundoscopic examination achieved full recovery of vision and this was statistically significant in our population. Nevertheless, the limited sample size of dogs with fundoscopic examination remains a limitation. Additionally, the involvement of multiple clinicians inevitably results in some degree of subjective outcome assessment. This is probably most prominent in the assessment of vision and degree of PLR deficit. In an attempt to decrease the degree of subjective PLR assessment, the result of this test was classified as reactive or non-reactive in our study. Accurate evaluation of vision in dogs is challenging. Future studies should combine behavioral assessment of visual acuity (151) and clinical testing with more objective methods such as visual evoked potentials (VEP). This is performed in people with ON to assess conduction recovery along the visual pathway and represents a valuable tool in interpreting relapses (152). The use of VEP in dogs has been reported, and results are reliable and reproducible (153–155). Other diagnostic modalities are also gaining interest in clinical and research settings in human medicine. Optical coherence tomography is a high-resolution imaging technology with applications in monitoring the course of MS and the severity of optic nerve damage (156, 157). This technique has been described in veterinary literature (158), but its clinical application in canine ON is still premature.

A high degree of confidence for the diagnosis of ON was reached in most of our cases. Nonetheless, considering the lack of histopathological confirmation of MUE, other differentials cannot be entirely ruled out, and this represents another limitation. The overlap between the clinical profiles of ON and ischemic optic neuropathy in human patients (159) could also occur in dogs, and this disorder should be considered as a differential in canine patients with sudden-onset blindness. Ischemic optic neuropathy was reported in a dog with acute onset pre-chiasmatic blindness and was diagnosed on MRI with diffusion-weighted (DWI) and apparent diffusion coefficient (ADC) map sequences. Additional findings of a mesencephalic infarct and hypertension were detected and supported the diagnosis (160). Diffusion-weighted and ADC map sequences were not performed in our study, which could represent a limitation. However, MRI and/or fundoscopic features of ON together with other clinicopathologic findings supported the diagnosis in our patients. Moreover, the presence of optic nerve abnormalities on routine MRI sequences is significantly associated with the diagnosis of ON rather than ischemic optic neuropathy in people (161, 162).

Therapeutic protocols varied between the studied dogs, and this is another limitation as it might have influenced the outcome. One could further argue that infectious diseases could have been missed as not all pathogens previously linked to ON were investigated. However, none of the included dogs deteriorated after immunosuppressive treatment. Infectious diseases are generally uncommon in our geographic region, and studies failed to identify infectious agents in CSF and brain samples of dogs with MUE (163–166). In contrast, sporadic evidence of specific pathogens has been reported in dogs with MUE, but their implication in the development of the disease warrants further investigation (165, 167–169).

In conclusion, our study identified prognostic factors that were associated with complete recovery of vision in 26 dogs with ON. A larger cohort of dogs is required to determine whether our findings are robust and whether any additional measurable parameters aid accurate prognostication. The validation of prognostic factors in canine ON could assist clinicians in improving current clinical practice. Finally, the apparent clinicopathologic and prognostic variability between different ON-associated diseases in people exposes the importance of developing an accurate etiopathogenic classification of distinct ON subtypes in dogs.

## Data Availability Statement

All datasets generated for this study are included in the article/supplementary material.

## Ethics Statement

The animal study was reviewed and approved by all institutions (Pride Veterinary Centre/University of Nottingham: 2274 180420; University of Liverpool: VREC664; Royal Veterinary College: URN SR2017-1191). Written informed consent was obtained from the owners for the participation of their animals in this study.

## Author Contributions

CP and AW designed and coordinated the study with the support of EB, MD, and IE. CP, SG, EB, AB, and IE provided all the necessary information from the clinical databases of the three participating referral hospitals. CP accessed and centralized all the clinical information. MD performed all the statistical analysis. CP processed the results and wrote the manuscript under the supervision and coordination of AW. All authors read and approved the final manuscript.

### Conflict of Interest

The authors declare that the research was conducted in the absence of any commercial or financial relationships that could be construed as a potential conflict of interest.

## Abbreviations

ON: optic neuritis
CNS: central nervous system
GME: granulomatous meningoencephalomyelitis
NLE: necrotizing leukoencephalitis
MUE: meningoencephalomyelitis of unknown etiology
PLR: pupillary light reflex
CSF: cerebrospinal fluid
TNCC: total nucleated cell count
CT: computed tomography
MRI: magnetic resonance imaging
ERG: electroretinography
FLAIR: fluid-attenuated inversion recovery
STIR: short tau inversion recovery
MUE-ON: MUE-associated ON
I-ON: isolated ON
mg: milligram
dl: deciliter
μl: microliter
kg: kilogram
SID: semel in die (once daily)
BID: bis in die (twice daily)
NMO: neuromyelitis optica
NMOSD: neuromyelitis optica spectrum disorders
MS: multiple sclerosis
GFAP: glial fibrillary acidic protein
AQP-4: aquaporin 4
MOG: myelin oligodendrocyte glycoprotein
ADEM: acute disseminated encephalomyelitis
OPC: oligodendrocyte precursor cells
OCB: oligoclonal bands
TG-2: transglutaminase 2
MOGAD: MOG-IgG-associated disorder.
